# Intelligent Reading of English Text Based on the Generative Model Constraint Label Fusion

**DOI:** 10.1155/2022/6728784

**Published:** 2022-09-21

**Authors:** Hua Yang, Huiliang Wei

**Affiliations:** ^1^College of Excellence, Baoding University of Technology, Baoding, Hebei, China; ^2^Hebei College of Science & Technology, Baoding, Hebei, China

## Abstract

The intelligent reading of English text is affected by complex environmental factors, which will result in low reading accuracy and poor reader experience. Based on the artificial intelligence model, this study constructs the artificial intelligence English text reading model by using the generative model constraint label, which helps to improve the intelligence of the English text reading effect. This study also designs a multigraph label fusion algorithm based on generative model constraints. By making full use of the prior knowledge of multiple graphs, the result of fusion graph segmentation is achieved. Moreover, this study also uses the combination of two algorithms, namely, the combination of GMM and MRF, to express the spatial correlation of local statistical features and image pixels in a comprehensive and all-round way. Another model design also includes a series of joint distributions of the learning data for the construction of the image energy function, and the conditional probability distribution is used as the model for prediction. At the end of the study, another variable control experiment is carried out to analyze the performance of the model and the accuracy of the model in English text recognition and classification is studied and counted. The research results show that the intelligent reading model constructed based on this study can meet the needs of the actual situation.

## 1. Introduction

As the third wave of artificial intelligence swept across, artificial intelligence technology, which is at the forefront of science and technology, began to penetrate, influence and be applied to all fields of society [1]. This includes the field of mobile reading. In addition to reading newspapers, news, and other content, people usually choose a book reading application software loaded on smart devices (such as mobile phones), such as palm reading, book banners, and novels [2]. In the field of artificial intelligence technology, intelligent computer technologies such as speech recognition technology, big data technology, and human-computer interaction are constantly developing and integrating. Product innovation through the development and application of the abovementioned intelligent technologies is a new channel for mainstream mobile reading applications to seek technological innovation [3]; providing users with multiple experiences that include audiovisual and even interaction in addition to single text reading has become a new focus of mobile reading app product development [4].

After entering the twenty-first century, our life belt is being changed by digital media, and we are in the era of transition from paper media to electronic media. This situation is reflected in reading, and browsing computer web files, reading emails, text messages, news on web pages, and comments have become very common on mobile phones and other electronic devices [5]. These behaviors are all digital reading. In the past ten years, many users have read this way. With the advancement of science and technology, reading behavior has undergone a quiet and dramatic change, and books have gradually developed in the direction of digitization, and e-books were born under this background [6]. Moreover, in order to read electronic books more conveniently, people have begun to use various electronic devices. However, compared with paper books, the most significant difference between e-books is the form and operation method and e-books should not be a substitute for the former [7]. People's reading carrier has undergone tremendous changes from traditional paper to mobile devices. However, the interface design of e-books has not exerted the maximum value of information technology and cannot provide sufficient support for communication between people. Moreover, with the increasing advancement of information technology, paper media have also embarked on the road of digitization and the interactive design mode of e-books urgently needs to be innovated [8]. At present, most of the reading software on the market basically simulates paper books, which redesigns the electronic reading interface to satisfy people's reading behavior. Because of this, this kind of simple imitation of paper books cannot make today's readers feel a better reading experience, and it often leads to various problems and also it does not allow users to browse data conveniently and comfortably. The root cause of this problem is that it is not people-oriented and it does not combine science and technology with people's reading methods, and blindly copy the results of the paper-based reading mode [9].

## 2. Related Work

In the past two years, the research on machine reading comprehension tasks has gained unprecedented attention abroad. Many well-known research institutions, such as Stanford University, Carnegie Mellon University, and Allen Research Institute, and industry giants such as IBM, Google, Facebook, and other giants, have also joined the research of this task.

The literature proposed two naive unsupervised baseline methods when publishing the MCTest dataset, which obtained the corresponding 4 paragraphs of text by concatenating the question and the candidate's answer [10]. For each piece of spliced text, this method uses a sliding window with the same length as the spliced text to slide in the reading material. Moreover, it counts the number of words in the window that contain the same words as the text to measure the degree of semantic matching between the question answer text and the reading material and then selects the answer. The literature added linguistic features such as co-referencing rules and negative detection to improve the performance of the system [11]. The literature used the rhetorical structure theory (RST) and the event entity co-referencing method to model multiple pairs of sentences [12]. Moreover, it obtained the part related to the question and made an organic combination to judge the implication relationship between the reading document and the question-answer text, and it also formalized the reading comprehension task into text implication for processing. Although there are differences in the internal structures of the three deep learning models involved in the literature, their overall framework is to carry out intelligent learning of the problem and the representation of each word in the original text through neural networks and to meet various types of reading needs of readers through diverse data accumulation and interaction [13]. According to the representation of the word, the final word with the highest score is the correct answer. Literature mentions that character-level word vectors and character-level dynamics can be combined by the gating mechanism design, which to some extent alleviates the problems caused by unregistered words [14]. Now, it has become a common practice in the field of machine reading comprehension to use both character-level and word-level word vectors to represent words together.

## 3. Atlas Preprocessing

**Figure 1:**
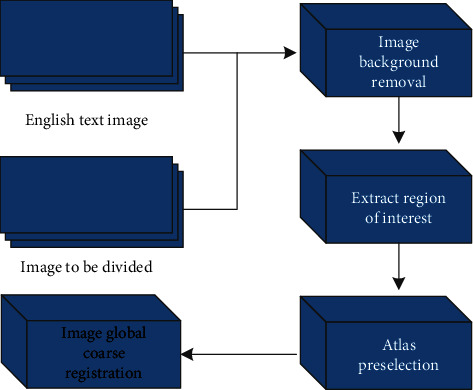
Flowchart of map preprocessing.

Figure 1 shows the steps of map preprocessing. In the Atlas preprocessing stage, each Atlas image and the image to be segmented need to be processed as follows: skull removal, extraction of regions of interest, Atlas preselection, and global coarse registration of Atlas images.

This article uses the brain surface extractor (BSE) algorithm to preprocess English text images. The BSE algorithm uses the Laplacian of Gaussian (LoG) edge detection operator for skull edge detection. This method uses a Laplacian sharpening filter and a Gaussian smoothing filter. The two filters are combined. It first performs the Gaussian filtering on the image to reduce noise while smoothing the image and then calculates the Laplacian second derivative. The edge of the skull in the image is obtained by zero crossings, and finally, the background is eliminated without changing the internal structure of the brain according to the knowledge of morphology.

The Gaussian function is as follows:

(1) $$\begin{array}{c} h\left(r\right)=-e^{-\frac{r^{2}}{2\sigma^{2}}}\text{.} \end{array}$$

Among them,

(2) $$\begin{array}{c} r^{2}=x^{2}+y^{2}\text{.} \end{array}$$

*σ* is the standard deviation. The Laplacian of this function (the second derivative of *r*) is as follows:

(3) $$\begin{array}{c} \nabla^{2}h\left(r\right)=-\left[\frac{r^{2}-\sigma^{2}}{\sigma^{4}}\right]e^{-\frac{r^{2}}{2\sigma^{2}}}\text{.} \end{array}$$

The image gradient algorithm takes into account the gray changes in the neighborhood of each pixel. It obtains the change rule by solving the first or second derivative between the pixel values of the neighborhood, that is, when the gray value of a neighborhood in the image changes greatly, the neighborhood must have a larger gradient value, which has edge features. Otherwise, the gray value of the neighborhood changes little and the image is smoother.

Mutual information can describe the correlation between two different systems. The greater the mutual information value, the greater the correlation between the two systems, or the more mutual information between the two systems. In English text image segmentation, since each Atlas image contains the same brain structure anatomy information, when the spatial positions of the two images are the same, the mutual information value of the corresponding pixel grayscale is the largest. The mutual information calculation formula is as follows:

(4) $$\begin{array}{c} I\left(F,M\right)=H\left(F\right)+H\left(M\right)-H\left(F,M\right)\text{,}\\ H=\overset{N}{\underset{i=0}{\sum }}p_{ij}\log p_{ij}\text{.} \end{array}$$

In the formula, *H*(*F*), *H*(*M*) represents the entropy of fixed image *F* and floating image *M*, *H*(*F*, *M*) is the mutual information of *F*, *M*.

(5) $$\begin{array}{c} p_{ij}=\frac{f\left(i,j\right)}{N^{2}}\text{.} \end{array}$$

Because the English text images are affected by instrument errors and the surrounding environment during the acquisition process, the differences between different images are large. Therefore, the global rigid coarse registration can be performed before the fine registration. The registration methods of English text images are classified according to the spatial transformation relationship and two categories of rigid registration and nonrigid registration can be obtained. Rigid registration is the overall translation, rotation, and scaling of the English text image, and both affine transformation and rigid body transformation belong to rigid registration. Nonrigid registration is achieved by calculating the position offset (deformation field) of each spatial point. This kind of registration method is computationally expensive and the spatial transformation is relatively complicated, but it can achieve better registration results. Nonrigid registration mainly includes Demons registration and B-spline interpolation transformation.

Due to the large amount of data in magnetic resonance images, rigid registration is not only computationally intensive but also time-consuming. Therefore, this study uses resampling to process the image to achieve the same effect as the coarse registration. The essence of resampling is image registration based on gray values, which can achieve the same effect as coarse registration. It aligns the Atlas grayscale image with the target image, and the sampling size of the image, the pixel spacing of the sampling space, and the direction of the three-dimensional image are all kept consistent. Moreover, resampling does not need to calculate the registration result as accurately as rigid registration, which reduces the calculation time. Therefore, this study uses bilinear interpolation in rough image registration. It maps the sampled image according to the distance, origin, and direction of the reference image to adjust the size and sample point of the two to be consistent. The core idea of bilinear interpolation is to perform linear interpolation in two directions. If it is assumed that *A*, *B*, *C*, and *D* are the four pixel points in the image; the coordinates are, respectively, marked as (*x*_1_, *y*_2_), (*x*_2_, *y*_2_), (*x*_1_, *y*_1_), an d(*x*_2_, *y*_1_), and *X* is the sampling point; then, the pixel value of the sampling point can be calculated by the following formula:

(6) $$\begin{array}{c} f\left(X_{1}\right)=\frac{x_{2}-x}{x_{2}-x_{1}}f\left(A\right)+\frac{x-x_{1}}{x_{2}-x_{1}}f\left(B\right)\text{,}\\ f\left(X_{2}\right)=\frac{x_{2}-x}{x_{2}-x_{1}}f\left(C\right)+\frac{x-x_{1}}{x_{2}-x_{1}}f\left(D\right)\text{,}\\ f\left(X\right)=\frac{y_{2}-x}{y_{2}-y_{1}}f\left(X_{1}\right)+\frac{y-y_{1}}{y_{2}-y_{1}}f\left(X_{2}\right)\text{.} \end{array}$$

**Figure 2:**
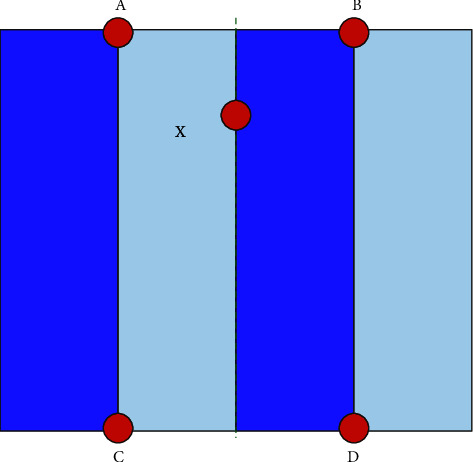
Bilinear interpolation principle diagram.

In the formula, *f*(*X*_1_) and *f*(*X*_2_) are pixel values linearly interpolated in the *x*-axis and *y*-axis directions, respectively, using four pixels around the sampling point. The bilinear interpolation principle diagram is shown in Figure 2.

The target energy function of the differential homeomorphism Demons registration algorithm is as follows:

(7) $$\begin{array}{c} E^{D_{i}}={\left\|F-M\circ S\circ e^{u}\right\|}^{2}+\frac{\sigma_{i}^{2}}{\sigma_{x}^{2}}dist{\left(S,S\circ e^{u}\right)}^{2}\text{.} \end{array}$$

In the formula, F represents the target image, *M* represents the floating image, and ∘ is the transformation operation. At the same time, *S* : *p*⟶*S*(*p*) is a nonparameter geometric transformation, which means that the target image and the floating image are geometrically transformed to achieve a mapping relationship, that is, the point *S*(*p*) in the floating image is the mapping value of the point *p* in the target image. *p* is the position point in the image, and *dist*(·)^2^ is the Euclidean distance. u is the updated deformation field and can also be called the deformation vector. When updating the deformation field, the exponential mapping of the deformation field and the compound operation *c* ← *S*∘*e*^*u*^ are used to obtain the following updated dense velocity field:

(8) $$\begin{array}{c} u\left(P\right)=-F\left(P\right)-M\circ S\left(p\right){\left\|J^{P}\right\|}^{2}+\frac{\sigma_{i}^{2}\left(p\right)}{\sigma_{x}^{2}}J^{P^{T}}\text{.} \end{array}$$

In the formula, *σ*_*i*_(*p*)=|*F*(*P*) − *M*∘*S*(*P*)| is the local estimation of image noise and *σ*_*x*_ represents the degree of uncertainty between *S* and *c*. The smaller the deformation, the smaller the value of *σ*_*x*_. The constraint condition of *σ*_*x*_ on the maximum step size ‖*u*(*P*)‖ of the dense velocity field is ‖*u*(*P*)‖ ≤ *σ*_*x*_2 , and adding this constraint can improve the image registration effect. *J*^*P*^ is the Demons force of diffeomorphism.

(9) $$\begin{array}{c} J^{P}=-\frac{1}{2}\left(\nabla_{P}^{T}F+\nabla_{P}^{T}\left(M\circ S\left(p\right)\right)\right)\text{.} \end{array}$$

Thus, the calculation efficiency of the algorithm can be improved.

The steps of the Demons algorithm are as follows:

  Step1: Demons deformation field *wW* is calculated and updated;
  Step2: u is subjected to fluid regularization, that is, *u* ← *K*_*fluid*_*∗u*
  Step3: *e*^*u*^ obtained by Lie group transformation is calculated and *c* ← *S*∘*e*^*u*^ is also calculated
  Step4: *S* is regularized by diffusion, *S* ← *K*_*diff*_*∗S* Markov characteristics describe the distribution characteristics between random variables, that is, when a random variable sequence is arranged in a time sequence, the value of the random variable at the current moment determines the distribution characteristic at the next moment, and its value is different from other moments. The Markov random field is cited in the image field, which means that the feature of any pixel in the image is only related to the pixel value of a small neighborhood where the pixel is located, and it will not be affected by other neighborhood pixel values. Combining the prior knowledge of the image with the neighborhood correlation of the MRF can effectively segment the MR image.

Image segmentation can be regarded as a label classification problem. The pixel set of the image to be segmented is denoted as *X*={*x*_1_, *x*_2_, ⋯, *x*_*n*_}, n is the number of pixels of the image, the image pixels belonging to a certain label category is denoted as *L*={*l*_1_, *l*_2_, ⋯, *l*_*m*_}, and *m* is the number of label categories. According to Bayesian estimation, the calculation of the image segmentation problem can be expressed as follows:

(10) $$\begin{array}{c} p\left(L\left|X\right.\right)=\frac{p\left(X\left|L\right.\right)\cdot p\left(L\right)}{p\left(X\right)}\text{.} \end{array}$$

In the formula, *p*(*L*) represents the prior probability of the image and *p*(*X*) is the probability density function of the image to be segmented, which is an invariant. Therefore, the maximum posterior probability of the abovementioned formula can be expressed as follows:

(11) $$\begin{array}{c} p\left(L\left|X\right.\right)=argmaxp\left(X\left|L\right.\right)\cdot p\left(L\right)\text{.} \end{array}$$

That is, only the maximum value of the numerator in formula (10) needs to be solved.

## 4. The Gaussian Mixture Model

For English text images, the gray level changes in each area of the image are relatively slow, and the overall gray level statistical histogram of the English text image always presents a multimodal distribution. Therefore, it can be described by a Gaussian mixture model (GMM). The GMM refers to a model formed by a linear combination of multiple Gaussian probability density functions. Generally speaking, GMM can fit most types of data distributions. This model is often used to describe a dataset containing multiple types of distributions or distributions with the same type of distribution but different parameters.

GMM has better results than a single Gaussian model in reducing the misclassification of pixels. Therefore, this article uses GMM to describe the grayscale characteristics of English text images, and its mathematical expression can be expressed by a weighted function of the probability distribution density function of a single Gaussian model as follows:

(12) $$\begin{array}{c} p\left(x_{i}\right)=\overset{m}{\underset{g=1}{\sum }}\alpha_{g}N_{g}\left(x_{i};\mu_{g},\varepsilon_{g}\right)\text{.} \end{array}$$

### 4.1. The Graph Cuts Algorithm

The graph cuts algorithm can be used to minimize the energy function. This algorithm can obtain a better segmentation effect while ensuring the segmentation speed. The graph cuts algorithm first maps the image to a weighted undirected graph *G*=(*V*, *E*). *G* contains source point *S* (representing target), sink point *T* (representing background), and node (pixel). *V* represents the set of image pixel nodes, *x*_*i*_ ∈ *V* is a pixel of the image, and adjacent nodes are connected by edges. At the same time, *E* represents the set of edges between nodes and *B*_*i*,*j*_ represents the connection weight of each edge of adjacent pixels, that is, the cost of cutting the edge. *R*_*i*_ represents the weight of the edge connecting each node with *S* and *T*. The weight represents the probability that the current node belongs to the target or background.

We assume that the label of each pixel in the image is *L*= and Zf is the background or foreground (target), and the calculation formula for the weight of the connecting edge of adjacent pixels is as follows:

(13) $$\begin{array}{c} B_{i,j}\left(x,l\right)=\lambda B_{\langle i,j\rangle }\delta \left(i,j\right)\text{.} \end{array}$$

In the formula,

(14) $$\begin{array}{c} \begin{array}{c} \lambda =50\\ B_{\langle i,j\rangle }=e^{-\left(\beta {\left\|z_{i}-z_{j}\right\|}^{2}\right)}\\ \delta \left(i,j\right)=\left\{\begin{array}{c} 1,x_{i}=x_{j}\\ 0,x_{i}\neq x_{j} \end{array}\right. \end{array}\text{.} \end{array}$$

*β* is determined by the degree of difference between image pixels as follows:

(15) $$\begin{array}{c} \beta ={\left(2{\left(z_{i}-z_{j}\right)}^{2}\right)}^{-1}\text{.} \end{array}$$

The calculation formula for the connection weight between each pixel and the source point and sink point is as follows:

(16) $$\begin{array}{c} R_{i}\left(x,l\right)=-\log\;p\left(l_{i},x_{i}\right)\text{.} \end{array}$$

This formula represents the negative logarithm of the probability that a pixel *x*_*i*_ is divided into a marker *l*_*i*_.

## 5. The Graph Cuts Label Fusion Algorithm Based on the Generative Model Constraints

This study uses the Gaussian mixture model to describe the distribution of image pixels. Combined with the neighborhood correlation of MRF, a graph cuts algorithm based on the generative model constraints is proposed, which improves the situation that the traditional label fusion methods cannot effectively describe the real model of the target structure. This algorithm is essentially a segmentation method that minimizes the energy function. The combination of GMM and MRF as generative models can effectively characterize the spatial correlation and local statistical characteristics of image pixels. By learning the joint distribution of the data, the conditional probability distribution is solved as a predictive model and the energy function is constructed. The graph cuts algorithm is used to correspond the energy function to the minimum cut set of the image, that is, the generative model is used to constrain the graph cuts algorithm to achieve the segmentation of the target image, and the energy function can be minimized by obtaining the minimum cut, that is, the segmentation is completed task.

*p*(*X*|*L*) · *p*(*L*) is *p*(*X*|*L*) which represents the joint probability distribution of image and segmentation information, so the formula can be written as follows:

(17) $$\begin{array}{c} p\left(L\left|X\right.\right)=argmaxp\left(X\left|L\right.\right)\text{.} \end{array}$$

According to the Hammersley–Clifford theorem, it is known that the Markov random field and the Gibbs distribution are consistent, that is, the probability distribution of the Markov random field can be represented by the Gibbs energy function (potential energy of the potential group) as shown

(18) $$\begin{array}{c} p\left(L\right)=z^{-1}e^{-u_{1}\left(L\right)}\text{.} \end{array}$$

Among them, *z*=∑_*L*_*e*^−*u*_1_(*L*)^ is the normalization constant and the parameter *X* can control the shape of *p*(*L*). The larger the *X* value, the smoother the *p*(*L*). *u*_1_(*L*)=∑_*c*_*v*_*c*_(*L*_*c*_) represents the prior energy function, where *c* is the set of all potential groups. *v*_*c*_(*L*_*c*_) represents the potential energy of the potential group, and the potential energy function is as follows:

(19) $$\begin{array}{c} v_{c}\left(L_{c}\right)=v_{i,j}\left(L_{i},L_{j}\right)=\left\{\begin{array}{cc} -\beta & L_{i}=L_{j}\\ \beta & L_{i}\neq L_{j} \end{array}\right.\text{.} \end{array}$$

Among them, *i* and j represent two adjacent pixels and *β* represents the coupling coefficient, which is used to describe the interaction strength between pixels in the potential cluster.

With the introduction of GMM, it is possible to judge which classification a certain pixel is in according to its value. The energy function of the likelihood function *p*(*X*|*L*) can be expressed by the following formula:

(20) $$\begin{array}{c} p\left(X\left|L\right.\right)=z^{-1}e^{-u_{2}\left(X\left|L\right.\right)}\text{,}\\ u_{2}\left(X\left|L\right.\right)==\underset{s}{\sum }\left(In\left(\sqrt{2\pi }\sigma_{i}\right)-In\alpha +\frac{{\left(X_{i}-\mu_{i}\right)}^{2}}{2\sigma_{i}^{2}}\right)\text{.} \end{array}$$

Among them, *Inα* is the weighting coefficient of the Gaussian mixture model, and *σ*_*i*_ and *μ*_*i*_ correspond to the variance and mean of the same labeled pixel set in the labeled set *L*, respectively.

The joint formula obtained is displayed as follows:

(21) $$\begin{array}{c} p\left(L\left|X\right.\right)=argmax\left\{z^{-1}e^{-\left(u_{2}\left(X\left|L\right.\right)+u_{1}\left(L\right)\right)}\right\}\text{.} \end{array}$$

We set the following:

(22) $$\begin{array}{c} R=argmax\left\{z^{-1}e^{-\left(u_{2}\left(X\left|L\right.\right)+u_{1}\left(L\right)\right)}\right\}\text{.} \end{array}$$

and we obtain the following:

(23) $$\begin{array}{c} InR\propto -\left(u_{2}\left(X\left|L\right.\right)+u_{1}\left(L\right)\right)\text{.} \end{array}$$

Thus, we derive as follows:

(24) $$\begin{array}{c} p\left(L\left|X\right.\right)=argmax\left(u_{2}\left(X\left|L\right.\right)+u_{1}\left(L\right)\right)\text{.} \end{array}$$

Thus, the problem of maximum posterior probability is transformed into a problem of solving the minimum value of the energy function.

## 6. Model Building

**Figure 3:**
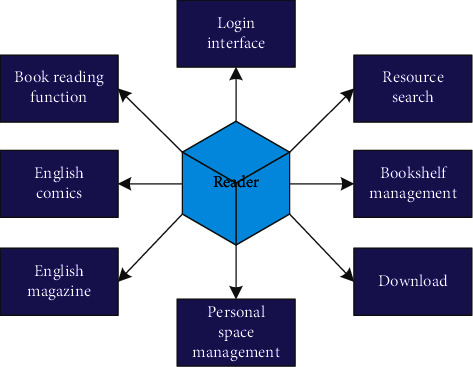
The reader function module diagram.

The reader starts with an English text reading service. The business process runs through reader DRM authentication, MEB e-book content analysis, reading resource content reading, and radiates to other auxiliary functions of each business module, such as bookmarking, book downloading, and automatic playback. The full-function module of the reader is shown in Figure 3. Due to the numerous business functions of the reader, the content of this study cannot describe all business function modules one by one. Therefore, this article only provides a relevant introduction to the content designed for other key business requirements derived from the core business.

**Figure 4:**
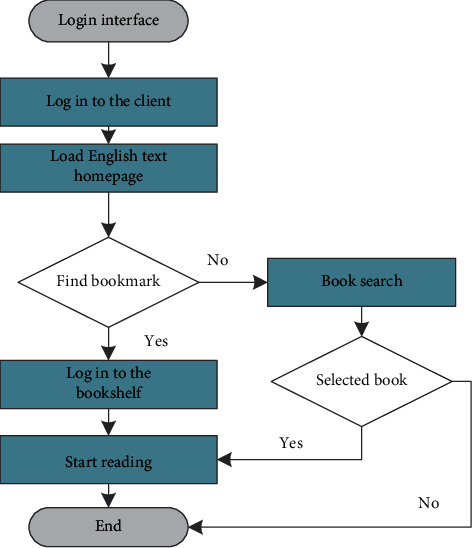
Bookmark function flowchart.

The function of bookmarks is to help users enter the previously reserved reading interface as soon as possible. The bookmark function is added to the bookshelf module, which makes it convenient for users to find the content of the books that need to be read before. The flow of the bookmark function design is that when users log in to the book reading module, if they need to read the relevant content previously read, they enter the interface where the bookmark function is located, and through the bookmark search, they enter the previously read interface. If there is no bookmark function and there is a need to read the previous content, the user must find the corresponding book through search, and find the corresponding chapter content within the scope of the book. The flowchart of the bookmark function is shown in Figure 4.

If the system is often in a reading background, the user will feel visual fatigue, so the background can be set in the black and white sky mode and the color difference can be adjusted in the book reading page. According to the current reading conditions, users can choose a background color that they feel comfortable to read books. This helps users to protect their eyesight, improve reading comfort, and also helps to extend the reading time. There are different groups of people using this system, so everyone has different vision conditions. In the process of reading, some users have blurred and unclear reading of the initial default font. Therefore, the font adjustment function is added to the reading page of the book. The font adjustment function can change the font size of the reading content. Users can choose a font suitable for their own reading for convenient reading.

The book recommendation function allows users to recommend favorite books they have read to users who have installed a smartphone reading system. After receiving the message, the other party will check the recommended books. This not only increases the market share of the smartphone reading system but also improves the stickiness of the system. It also facilitates users to obtain more up-to-date and useful information, which achieves a win-win situation for service providers and users.

**Figure 5:**
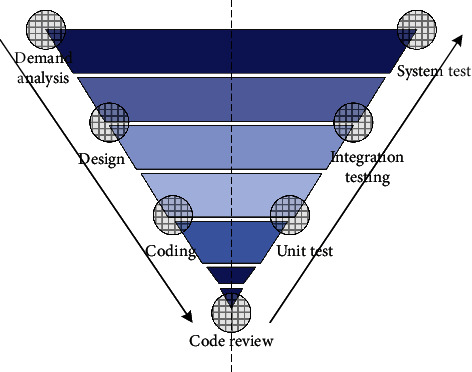
V-type test model diagram.

Software testing is the guarantee of software quality, so the testing process adopts the V-graph model for testing, as shown in Figure 5. The V-shaped diagram of the development model is accompanied by the entire software development cycle, and the object of testing is not only the program but also the requirements and designs. The V-shaped chart development model is conducive to the early and comprehensive discovery of problems. In the software development stage, the development process needs to be from rough to detailed, and it is necessary to adopt advanced requirements analysis, requirement decomposition, and list all requirements to ensure that the required information is clear and not omitted. Then, we design each requirement. The design includes system design, outline design, and detailed design. It also adheres to the principle of gradual progress from coarse to fine to make the design clearer and simpler. This development model is also a typical waterfall development model but we use agile development, which belongs to the waterfall model applied in the iterative model.

**Figure 6:**
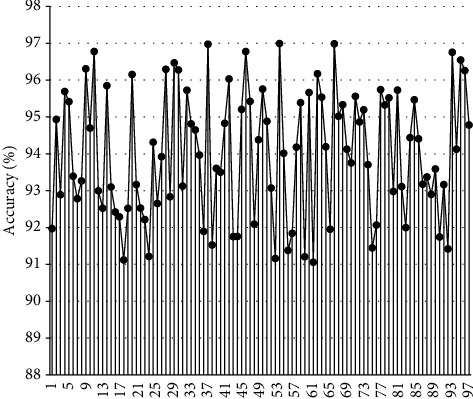
Accuracy of English reading.

Based on the abovementioned construction model, the model is tested and the reading situation of English text is counted. This article first sets up100 groups of English reading texts to analyze the accuracy of English reading recognition. The results are shown in Figure 6.

**Figure 7:**
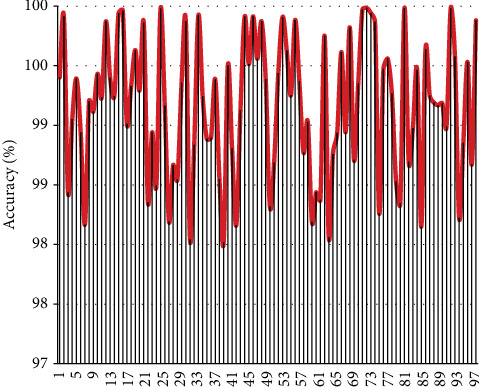
A statistical diagram of the model's recognition accuracy of English text types.

The data show that the intelligent reading model constructed in this study has a high reading accuracy for English texts. The recognition effect of English text types in the system is shown in Figure 7.

From the results, it can be seen that the model constructed in this study has a very good classification effect on English text.

## 7. Conclusion

In order to recognize the intelligent reading of English text at the image level, this study proposed a multi-image label fusion algorithm design model of graph cuts based on the generative model constraints. This model can be used for intelligent and automatic pixel segmentation of the image structure of English text, which is the result of unitized segmentation of the fused image by utilizing the prior knowledge data reserve of multiple images. In addition, in order to reduce the complexity of the subsequent calculation, this study uses the open source code library 1 TK and visual studio-integrated development environment to process the image in advance in the image preprocessing module.

This study proposes to use a generative model combining GMM and MRF to express the spatial correlation and local statistical characteristics of image pixels. By learning the joint distribution of the data, the conditional probability distribution is solved as a prediction model and the image energy function is constructed. Moreover, this study uses the graph cuts algorithm to correspond the energy function to the minimum cut set of the image, that is, it uses the generative model to constrain the graph cuts algorithm to obtain the minimum cut to minimize the energy function. In addition, after constructing the model structure, this article sets up a control experiment to analyze the model's performance from two aspects: the accuracy of the model's recognition of English text and the accuracy of text classification. The research results show that the intelligent reading model constructed in this study performs well in meeting the needs of practical applications.

## Data Availability

The data supporting the current study are available from the corresponding author upon request.
